# Verification of Quantitative Hyperproperties Using Trace Enumeration Relations

**DOI:** 10.1007/978-3-030-53288-8_11

**Published:** 2020-06-13

**Authors:** Shubham Sahai, Pramod Subramanyan, Rohit Sinha

**Affiliations:** 8 Microsoft Research Lab, Redmond, WA USA; 9 University of Southern California, Los Angeles, CA USA; 10 Indian Institute of Technology, Kanpur, India; 11 Visa Research, Palo Alto, USA

## Abstract

Many important cryptographic primitives offer probabilistic guarantees of security that can be specified as quantitative hyperproperties; these are specifications that stipulate the existence of a certain number of traces in the system satisfying certain constraints. Verification of such hyperproperties is extremely challenging because they involve simultaneous reasoning about an unbounded number of different traces. In this paper, we introduce a technique for verifying quantitative hyperproperties based on the notion of trace enumeration relations. These relations allow us to reduce the problem of trace-counting into one of model-counting of formulas in first-order logic. We also introduce a set of inference rules for machine-checked reasoning about the number of satisfying solutions to first-order formulas (aka model counting). Putting these two components together enables semi-automated verification of quantitative hyperproperties on infinite-state systems. We use our methodology to prove confidentiality of access patterns in Path ORAMs of unbounded size, soundness of a simple interactive zero-knowledge proof protocol as well as other applications of quantitative hyperproperties studied in past work.

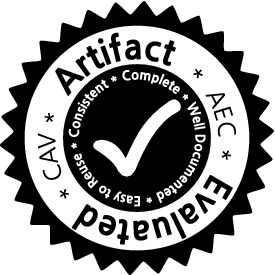



## Introduction

Recent years have seen significant progress in automated and semi-automated techniques for the verification of security requirements of computer systems [4, 10, 16, 19, 30, 47, 50, 55]. Much of this progress has built on the theory of *hyperproperties* [21], and these have been used extensively in analysis of whether systems satisfy secure information flow properties [1, 2, 6, 8, 15, 28, 35, 37, 39, 49, 57] such as observational determinism [41, 55] and non-interference [32]. Unfortunately, the security specification of several important security primitives cannot be captured by secure information flow properties like observational determinism. In particular, observational determinism and non-interference are not applicable when reasoning about algorithms that offer probabilistic – as opposed to deterministic – guarantees of confidentiality and integrity. Prominent examples of security primitives offering probabilistic guarantees include Path ORAM [48] and various zero-knowledge proof protocols.

A promising direction for the verification of such protocols are the class of quantitative hyperproperties [29], one example of which is deniability [12, 14]. Deniability states that for every infinitely-long sequence of observations that an adversary makes, there are (exponentially) many different secrets that could have resulted in exactly these observations. Therefore, the adversary learns very little about the secrets in an execution from a particular sequence of observations.

How does one prove a quantitative hyperproperty like deniability? Suppose our goal is to show that for every trace of adversary observations, there exist $2^{n}$ traces with the same observations but different secrets. Here *n* is a parameter of the system, e.g., the length of a password in bits. One option, first suggested by Yasuoka and Terauchi [54] and recently revisited by Finkbeiner, Hahn, and Torfah [29], is to consider the following *k*-trace property, where $k=2^{n}+1$.

$$\begin{aligned} \forall&\pi _{0}.~\exists \pi _{1}, \pi _{2}, \dots , \pi _{2^{n}}.~&\\&\Big (\bigwedge _{j=1}^{2^{n}} obs (\pi _{0}) = obs (\pi _{j}) \Big ) \wedge \Big (\bigwedge _{j=1}^{2^{n}} \bigwedge _{k=1}^{2^{n}}~ (j \ne k) \Rightarrow secret (\pi _{j}) \ne secret (\pi _{k})\Big )&\end{aligned}$$

The property states that for every trace of the system, there must exist $2^{n}$ other traces with identical observations and pairwise different secrets. In the above, $\pi _{0}, \pi _{1}, \dots$ represent trace variables, $obs (\pi _{j})$ refers to the trace of adversary observations projected from the trace $\pi _{j}$, while $secret (\pi _{j})$ refers to the trace of secret values in the trace $\pi _{j}$. There are at least three problems with the verification of the above property. First, the size of this property grows exponentially with *n*; verification needs to reason about $2^n$ traces simultaneously and is not scalable. The second problem is quantifier alternation. Even if we could somehow reason about $2^n$ traces, we have to show that *for every* trace $\pi _{0}$, there *exist* $2^n$ other traces satisfying the above condition. The third problem is that the above technique does not work for *symbolic* bounds. While it is possible – at least in principle – to use the above construction by picking a specific value of *n*, say 16, to show that $2^{16}$ traces exist that satisfy deniability, we would like to show that the property holds for all *n*, where *n* is a state variable or parameter of the transition system. Capturing the dependence of the trace-count bound on parameters, such as *n*, is important because it shows that the attacker has to work exponentially harder as *n* increases. Such general proofs are not possible by reduction to a *k*-trace property because the construction requires *k* be bounded.

Recent work by Finkbeiner, Hahn, and Torfah [29] has made significant progress in addressing the first two problems by showing a reduction from *k*-trace property checking into the problem of maximum model counting [31]. However, their technique still produces a propositional formula whose size grows exponentially in the size of the quantitative hyperproperty. Further, model counting itself is a computationally hard problem that is known to be $\#P$-complete, and maximum model counting is even harder. As a result, their technique does not scale well and times out on the verification of an 8-bit leakage bound for an 8-bit password. Finally, their method does not support symbolic bounds, and therefore cannot be used to verify parametric systems; we verify several examples of such systems in this paper (e.g., Path ORAM [48] of symbolic size).

In this work, we propose a new technique for quantitative hyperproperty verification that addresses each of the above problems. Our approach is based on the following insights. First, instead of trying to count the number of traces that have the same observations and different inputs, we instead show injectivity/surjectivity from satisfying assignments of a first-order formula to traces of a transition system. This allows us to bound the number of traces satisfying the quantitative hyperproperty by the number of satisfying solutions to this formula. We introduce the notion of a trace enumeration relation to formalize this relation between the first-order formula and traces of the transition system. An important advantage of the above reduction is that proving the validity of a trace enumeration relation is only a hyperproperty – not a quantitative hyperproperty.

Next, we develop a novel technique to bound the number of satisfiable solutions to a first-order logic formula, which is of independent interest. While this is a hard problem, we exploit the fact that our formulas have a significant amount of structure. We introduce a set of inference rules inspired by ideas from enumerative combinatorics [13, 52, 56]. These rules allow us to bound the number of satisfying assignments to a formula by making only satisfiability queries.

In summary, our techniques can prove quantitative hyperproperties with symbolic bounds on parametric infinite-state systems. We demonstrate their utility by verifying representative quantitative hyperproperties of diverse applications.

**Contributions**

1. We introduce a specification language for quantitative hyperproperties (QHPs) over symbolic transition systems and define formal satisfaction semantics for this language. Our specification language is more expressive than past work on QHP specification because it allows the bound to be a first-order formula over the state variables of the transition system.
2. We provide several examples of QHPs relevant to security verification. We identify a new class of QHPs, referred to as soundness hyperproperties, applicable to protocols that provide statistical guarantees of integrity.
3. We propose a novel semi-automated verification methodology for proving that a system satisfies a QHP. Our methodology applies to properties that involve a single instance of quantifier alternation and works by reducing the problem of QHP verification to that of checking non-quantitative hyperproperties over two and three traces of the system and counting satisfiable solutions to a formula in first-order logic.
4. We introduce a set of inference rules for bounding the number of satisfiable solutions to a first-order logic formula, using only satisfiability queries.
5. We demonstrate the applicability of our specification language and verification methodology by providing proofs of security for Path ORAM, soundness of a simple zero-knowledge protocol, as well as examples taken from prior work on quantitative security specifications. We show that our verification methodology scales to larger systems than could be handled in prior work. To the best of our knowledge, our work is the first machine-checked proof of confidentiality of the access patterns in Path ORAM.

## Motivating Example

In this section, we first introduce the model of transition systems used in this paper. We then discuss quantitative hyperproperty (QHP) specification and verification for our running example – a simple zero-knowledge puzzle.

### Preliminaries

Let $FOL (\mathcal {T})$ denote first-order logic modulo a theory $\mathcal {T}$. The theory $\mathcal {T}$ is assumed to be multi-sorted, includes the theory of linear integer arithmetic (LIA), and contains the $=$ relation. Let $\varSigma _{\mathcal {T}}$ be the theory $\mathcal {T}$’s signature: the set consisting of the constant, function, and predicate symbols in the theory. We say that a formula is a $\varSigma _{\mathcal {T}}$-formula if it consists of the symbols in $\varSigma _{\mathcal {T}}$ along with variables, logical connectives, and quantifiers. We only consider theories which are such that the set of satisfying assignments for any $\varSigma _{\mathcal {T}}$-formula is a countable set.^1^

For every variable *x*, we will assume there exists a unique variable $x'$, which we refer to as the primed version of *x*. We will use *X*, *Y*, and *Z* to denote sets of variables. Given a set of variables *X*, we will use $X'$ to refer to the set consisting of the primed version of each variable in *X*, that is $X' = \{ x'~|~x \in X \}$. Similarly $X_1$, $X_2$, etc. are sets consisting of new variables defined as follows: $X_1 = \{ x_1~|~x \in X \}$ and $X_2 = \{ x_2~|~x \in X \}$. We will use *F*(*X*) to denote the application of a function or predicate symbol *F* on the variables in the set *X*. A satisfying assignment $\sigma$ to the formula *F*(*X*) is written as $\sigma \,\models \,F(X)$. Given a formula *F*(*X*) and a satisfying assignment $\sigma$ to this formula, we will denote the valuation of the variable $x \in X$ in the assignment $\sigma$ as ${\sigma }({x})$. We will abuse notation in two ways and also write ${\sigma }({X})$ to refer to a map from the variables $x \in X$ to their assignments in $\sigma$. We will also write ${\sigma }({G(X)})$ to denote the valuation of the term *G*(*X*) under the assignment $\sigma$.

The number of satisfiable assignments for the variables in the set *X* to a formula *F*(*X*, *Y*) as a function of the variables *Y* will be denoted by ${\#}{X}.\,{F(X, Y)}$. ${\#}{X}.\,{F(X, Y)}$ is the function ${\uplambda \mathrm {Y}~.~ |\{ {\sigma }({X}) ~|~ \sigma \,\models \,F(X, \mathrm {Y}) \}|}$ evaluated at *Y*; |*S*| is the cardinality of the set *S*. For example, consider the predicate $f(i, n) \doteq (0 \le i < 2n)$. In this case, ${\#}{i}.\,{f(i, n)} = \max {(0, 2n)}$, meaning that for a given value of $n > 0$, there are 2*n* satisfying assignments to *i*.

#### Definition 1 (Transition System)

A transition system *M* is defined as the tuple ${M{} = \langle X, Init {}(X), Tx {}(X,X') \rangle }{}$. *X* is a finite set of (uninterpreted) constants that represents the state variables of the transition system. $Init$ and $Tx$ are $\varSigma _{\mathcal {T}}$-formulas representing the initial states and the transition relation, respectively. $Init$ is defined over the signature $\varSigma _{\mathcal {T}}\cup X$. $Tx$ is over the signature $\varSigma _{\mathcal {T}}\cup X\cup X'$; *X* represents the pre-state of the transition and $X'$ represents its post-state.

A state of the system is an assignment to the variables in *X*. We use ${\sigma ^{0}}, {\sigma ^{1}}, {\sigma ^{2}}$ etc. to represent states. A trace of the system *M* is an infinite sequence of states ${\tau }_{} = {\sigma ^{0}}{\sigma ^{1}}{\sigma ^{2}}\dots$ ${\sigma ^{i}}$ $\dots$ such that $Init ({\sigma ^{0}})$ is valid and for all $i \ge 0$, $Tx ({\sigma ^{i}}, {\sigma ^{i+1}})$ is valid; in order to keep notation uncluttered, we will often drop the $\ge 0$ qualifier when referring to trace indices. We assume that every state of the transition system has a successor: for all ${\sigma ^{}}$ there exists some ${\sigma ^{}}'$ such that $Tx ({\sigma ^{}}, {\sigma ^{}}')$ is valid, ensuring every run of the system is infinite. We will represent traces by $\tau , \tau _1, \tau _2$, etc. Given a trace ${\tau }_{}$, we refer to its $i^{th}$ element by $\tau ^i$. If ${\tau }_{} = {\sigma ^{0}}{\sigma ^{1}}\dots$, then ${\tau _{}^{0}} = {\sigma ^{0}}$ and ${\tau _{}^{1}} = {\sigma ^{1}}$. The notation $\tau ^{[i,\infty ]}$ refers to the suffix of trace ${\tau }_{}$ starting at index *i*. The set of all traces of the system *M* is denoted by $\varPhi {}_{M}$. Given a state ${\sigma ^{}}$ and a variable $x \in X$, ${{\sigma ^{}}}({x})$ is the valuation of *x* in the state ${\sigma ^{}}$.

### Motivating Example: Zero-Knowledge Hats

Zero-knowledge (Z-K) proofs are constructions involving two parties: *a prover* and *a verifier*, where the prover’s goal is to convince the verifier about the veracity of a given statement without revealing any additional information. We motivate the need for quantitative hyperproperty verification using a Z-K puzzle.

**Puzzle Overview:** Consider the following scenario. Peggy has a pair of otherwise identical hats of different colors (say, yellow and green). She wants to convince Victor, who is yellow-green color blind, that the hats are of different colors, without revealing the colors of the hats. This problem can be solved using the following interactive protocol. Peggy gives both hats to Victor, and Victor randomly chooses a hat behind a curtain and shows it to Peggy. Next, he goes back behind the curtain and uniformly randomly chooses if he wants to switch the hat or not. He now appears in front of Peggy and asks: “Did I switch?”

If the hats are really of different colors, Peggy will be able to answer correctly with probability 1. If Peggy is cheating – the hats are in fact of the same color – her best strategy is to guess, and with probability 0.5 she will answer incorrectly. If the interaction is repeated *k*-times, Peggy will be caught with probability $1 - 2^{-k}$. The interaction between Peggy and Victor only reveals the fact that Peggy can detect a switch and not the color of the hat, making this zero-knowledge.

**Verification Objectives:** A zero-knowledge proof must satisfy three properties: *completeness* (an honest prover should be able to convince an honest verifier of a true statement), *soundness* (a cheating prover can convince an honest verifier with negligible probability) and *zero-knowledge* (no information apart from the veracity of the statement should be revealed). Completeness is a standard trace property, while zero-knowledge is the 2-safety property of indistinguishability. Consequently, the main challenge in automated verification of the zero-knowledge protocol described above is that of soundness. In this section, we discuss its specification and verification using quantitative hyperproperties.

**Fig. 1.**
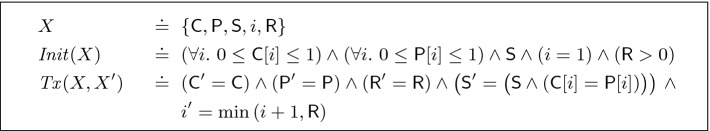
Transition system model of the example protocol.

**Soundness as a Quantitative Hyperproperty:** Consider the transition system ${M{} = \langle X, Init {}(X), Tx {}(X,X') \rangle }{}$, shown in Fig. 1, representing this protocol. The variable $\mathsf {R}{}$ is a *parameter* of the system and refers to the number of rounds of the protocol. $\mathsf {C}$ and $\mathsf {P}$ are boolean arrays representing the challenges from the verifier to the prover, and the responses from the prover to the verifier, respectively. *i* is the current round, and $\mathsf {S}{}$ is a boolean flag that corresponds to whether the zero-knowledge proof has succeeded. $\mathsf {C}$ and $\mathsf {P}$ are initialized non-deterministically to model the fact that the verifier chooses their challenges randomly, and a cheating prover’s best strategy is guessing. While a cheating prover can use any strategy, if the challenges are indistinguishable to her, then the best strategy is to sample responses from a uniform distribution.

Soundness is captured by the following quantitative hyperproperty (QHP):

1 $$\begin{aligned} \forall \pi _{0}.\# \pi _{1}\!\!:\!\mathbf{F }\,(\delta _{\pi _{j}, \pi _{k}}).~\mathbf{G }\,(\psi _{\pi _{0}, \pi _{1}}) ~\ge \,2^\mathsf {R}- 1 \end{aligned}$$

We will provide formal satisfaction semantics for QHPs in Sect. 3. For now, we informally describe its meaning. The term $\# \pi _{1}\!\!:\!\mathbf{F }\,(\delta _{\pi _{j}, \pi _{k}}).~\mathbf{G }\,(\psi _{\pi _{0}, \pi _{1}}) ~\ge \,2^\mathsf {R}{}-1$ introduces a counting quantifier which stipulates the existence of at least $2^\mathsf {R}- 1$ traces satisfying certain conditions: (i) these traces must all be pairwise-different, where difference is defined by satisfaction of the formula $\mathbf{F }\,(\delta _{\pi _{j}, \pi _{k}})$ and (ii) all of these traces must be related to trace $\pi _{0}$ by the relation $\mathbf{G }\,(\psi _{\pi _{0}, \pi _{1}})$.

The state predicates $\delta$ and $\psi$ are defined as follows.

$$\begin{aligned} \delta ({\sigma ^{}}_1, {\sigma ^{}}_2) ~\doteq ~&{{\sigma ^{}}_1}({\mathsf {P}[i]}) \ne {{\sigma ^{}}_2}({\mathsf {P}[i]})&\nonumber \\ \psi ({\sigma ^{}}_1, {\sigma ^{}}_2) ~\doteq ~&\big ({{\sigma ^{}}_1}({(i = \mathsf {R}) \Rightarrow \mathsf {S}}) \Rightarrow {{\sigma ^{}}_2}({(i = \mathsf {R}) \Rightarrow \lnot \mathsf {S}})\big ) ~~~\wedge \\&\big ({{\sigma ^{}}_1}({\mathsf {C}{}})= {{\sigma ^{}}_2}({\mathsf {C}{}}) \wedge {{\sigma ^{}}_1}({\mathsf {R}{}}) = {{\sigma ^{}}_2}({\mathsf {R}{}}) \big )&\nonumber \end{aligned}$$

**Fig. 2.**
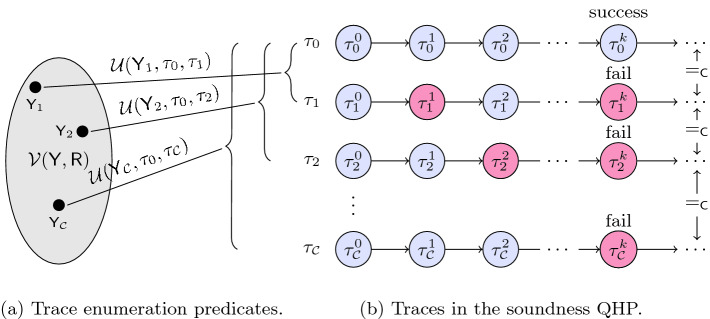
Using trace enumeration predicates to verify the soundness QHP.

The requirement imposed by $\delta$ is that Peggy’s responses be different at some step *i* for every pair of traces captured by the counting quantifier. $\psi$ says that if trace $\pi _{0}$ is a trace where Peggy’s cheating succeeds (i.e., $\mathsf {S}{} = true$ when $i = \mathsf {R}{}$), then in all traces captured by $\pi _{1}$, the challenges and number of rounds are the same as $\pi _{0}$ but Peggy’s cheating is detected by Victor (i.e., $\mathsf {S}= false$ when $i = \mathsf {R}{}$). These requirements are illustrated in Fig. 2(b).

The QHP requires that for every trace in which a cheating prover succeeds in tricking the verifier for a given trace of challenges, there are $2^\mathsf {R}{} - 1$ other traces with the same challenges in which the prover’s cheating is detected. Even though soundness is a probabilistic property over the distribution of the system’s traces, it can be reduced to counting (and thus specified as a QHP) because each execution trace is sampled uniformly from a finite set. Therefore, if the QHP is satisfied, Peggy’s probability of successful cheating is upper-bounded by $2^{-\mathsf {R}{}}$.

### Solution Outline

To prove a QHP of the form $\forall \pi _{0}.~\# \pi _{1}\!\!:\!\varDelta _{\pi _{j}, \pi _{k}}.~\varphi ~\triangleleft \,N(Z){}$, we construct a *trace enumeration predicate* $\mathcal {V}_{}({\mathsf {Y}, Z})$ and show an injective/bijective mapping from assignments to $\mathsf {Y}$ in $\mathcal {V}_{}({\mathsf {Y}, Z})$ and traces of the system. This allows us to prove $\forall \pi _{0}.~\# \pi _{1}\!\!:\!\varDelta _{\pi _{j}, \pi _{k}}.~\varphi ~\triangleleft \,{\#}{Y}.\,{\mathcal {V}_{}({Y, Z}){}}$. This part of the proof relies on the notion of a trace enumeration relation (Sect. 4). In the next step, we show that ${{\#}{Y}.\,{\mathcal {V}_{}({Y, Z}){}}} \triangleleft N(Z)$ using the inference rules presented in Sect. 5. We now describe these steps in the context of the motivating example.

*Verification of Soundness for the Z-K Hats Puzzle:* Property 1 is illustrated in Fig. 2(b). ${\tau }_{0}$ is a trace where the Z-K proof succeeds, while the proof fails for the set of traces $\varPhi {}_{\mathcal {C}}{} = \{ {\tau }_{1}, {\tau }_{2}, \dots , {\tau }_{\mathcal {C}{}} \}$. The red states show the particular step of the proof in which an incorrect response is given by the prover, and each of these steps as well as their associated prover responses are pairwise different. The QHP is satisfied if $|\varPhi {}_{\mathcal {C}}{}| \ge 2^\mathtt {R} - 1$ for every ${\tau }_{0} \in \varPhi {}_{M}$, where $\mathtt {R} = {{\tau _{0}^{0}}}({\mathsf {R}{}})$.

The first step in our methodology is to construct a parameterized relation, called a trace enumeration relation, ${\mathcal {U}(\mathsf {Y}, {\tau }_{0}, {\tau }_{1})}$. This relates ${\tau }_{0}$ to each trace in the set $\varPhi {}_{\mathcal {C}}{}$ and is parameterized by $\mathsf {Y}$. For every value of the parameter $\mathsf {Y}$, $\mathcal {U}$ relates a trace in which the proof succeeds (${\tau }_{0}$) to a trace in which the proof fails (${\tau }_{1}$). For every trace ${\tau }_{0}$ in which the proof succeeds, the set $\{ {\tau }_{1} ~|~ \exists \mathsf {Y}.~{\mathcal {U}(\mathsf {Y}, {\tau }_{0}, {\tau }_{1})} \}$ corresponds to the set of traces with the same challenges and the same number of rounds, but with failed proofs of knowledge. Note this is a subset of $\varPhi {}_{\mathcal {C}}{}$.

Next, we construct a predicate $\mathcal {V}_{}({\mathsf {Y}, \mathsf {R}{}})$ which defines valid assignments to $\mathcal {V}$ for a particular value of $\mathsf {R}{}$. For a particular $\mathsf {R}{}$, consider the set: $\{ \sigma (\mathsf {Y}) ~|~ \sigma \,\models \,\mathcal {V}_{}({\mathsf {Y}, \mathsf {R}{}}) \}$. Suppose we are able to show that the relation $\mathcal {U}$ is injective in $\mathsf {Y}$ and ${\tau }_{0}$ for assignments to $\mathsf {Y}$ drawn from this set, then we can lower-bound the size of $\varPhi {}_{\mathcal {C}}{}$ by the size of this set. In other words, we have reduced the problem of trace counting to the problem of counting assignments to $\mathcal {V}_{}({\mathsf {Y}, \mathsf {R}{}})$.

Precisely stated, using $\mathcal {V}$ and $\mathcal {U}$, we show the following.

1. For every trace ${\tau }_{0}$, and every assignment $\mathsf {Y}_i$ satisfying $\mathcal {V}_{}({\mathsf {Y}_i, {{\tau _{0}^{0}}}({\mathsf {R}{}})})$, there exists a corresponding trace ${\tau }_{i}$ that satisfies both ${\mathcal {U}(\mathsf {Y}_i, {\tau }_{0}, {\tau }_{i})}$ and $\psi ({\tau }_{0}, {\tau }_{i})$. (Note ${{\tau _{0}^{0}}}({\mathsf {R}{}})$ refers to the valuation of $\mathsf {R}{}$ in the initial state of ${\tau }_{0}$.)
2. Given two different satisfying assignments to $\mathcal {V}$ for a particular value of $\mathsf {R}{}$, say $\mathsf {Y}_j$ and $\mathsf {Y}_k$, the corresponding traces ${\tau }_{j}$ and ${\tau }_{k}$ are guaranteed to have different prover responses; in other words, the traces satisfy $\delta ({\tau }_{j}, {\tau }_{k})$.

The above two properties, illustrated in Fig. 2(a), imply there is an injective mapping from satisfying assignments of $\mathcal {V}_{}({\mathsf {Y}, \mathsf {R}{}})$ to traces in $\varPhi {}_{\mathcal {C}}{}$. Therefore, the number of traces in $\varPhi {}_{\mathcal {C}}{}$ can be lower bounded by the number of satisfying assignments to $\mathsf {Y}$ in $\mathcal {V}_{}({\mathsf {Y}, \mathsf {R}{}})$, i.e. ${\#}{\mathsf {Y}}.\,{\mathcal {V}_{}({\mathsf {Y}, \mathsf {R}{}})}$. We have reduced the difficult problem of counting traces into a slightly easier problem of counting satisfying assignments to a $FOL (\mathcal {T})$ formula.

The final step is to bound ${\#}{\mathsf {Y}}.\,{\mathcal {V}_{}({\mathsf {Y}, \mathsf {R}{}})}$. For example, one well-known idea from enumerative combinatorics is that if a set *A* is the union of disjoint sets *B* and *C*, then $|A| = |B| + |C|$. Translated to model counting, the above can be written as ${\#}{X}.\,{F(X, Y)}= {\#}{X}.\,{G(X, Y)} + {\#}{X}.\,{H(X, Y)}$ if $F(X, Y) \Leftrightarrow G(X, Y) \vee H(X, Y)$ is valid and $G(X, Y) \wedge H(X, Y)$ is $\mathsf {unsat}$.^2^ We present a set of inference rules in Sect. 5 that build on this and related ideas. These inference rules allow us derive a machine-checked proof of the bound ${\#}{\mathsf {Y}}.\,{\mathcal {V}_{}({\mathsf {Y}, \mathsf {R}{}})} \ge 2^{\mathsf {R}{}} - 1$, thus completing the proof of Property 1 for the Z-K hats puzzle.

## Overview of Quantitative Hyperproperties

This section introduces a logic for the specification of quantitative hyperproperties over symbolic transition systems. We present satisfaction semantics for this logic and then discuss its applications in security verification.

**Fig. 3.**
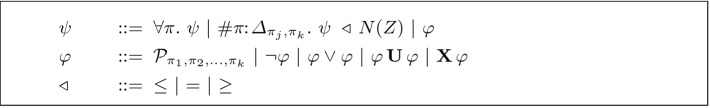
Grammar of Quantitative HyperLTL.

### Quantitative Hyperproperties

Figure 3 shows the syntax of Quantitative HyperLTL, our extension of HyperLTL [30] that allows specification of quantitative hyperproperties over symbolic transition systems. There are two noteworthy differences from the presentation of HyperLTL in [30]. The first is the predicate $\mathcal {P}_{\pi _{1}, \pi _{2}, \dots , \pi _{k}}$. This refers to a *k*-ary state predicate $\mathcal {P}{}$ that is applied to the first element of each trace in the subscript. These are analogous to atomic propositions in presentations that use Kripke structures and are defined as *k*-ary state predicates to capture relational properties over traces of the transition system. For example, consider the predicate $\mathcal {P}({\sigma ^{}}_0, {\sigma ^{}}_1) \doteq ( input ({\sigma ^{}}_0) = input ({\sigma ^{}}_1))$. Given this definition, a system $M{}$ with exactly two traces $\varPhi {}_{M{}} = \{ {\tau }_{1}, {\tau }_{2} \}$ satisfies the HyperLTL formula $\forall \pi _{1}, \pi _{2}.~\mathcal {P}_{\pi _{1}, \pi _{2}}$ iff $input ({\tau _{1}^{0}}) = input ({\tau _{2}^{0}})$. This hyperproperty requires that the input in the initial state of the system be deterministically initialized.

The second difference is the new *counting quantifier*: $\# \pi _{}\!\!:\!\varDelta _{\pi _{j}, \pi _{k}}.~\psi ~\triangleleft \,N(Z)$.^3^ $\varDelta _{\pi _{j}, \pi _{k}}$ is an unquantified HyperLTL formula over two “fresh” trace variables $\pi _{j}$ and $\pi _{k}$ that encodes when two traces are considered different. $\psi$ is another (possibly-quantified) HyperLTL formula. The operator $\triangleleft$ can be $\le$, $=$, or $\ge$. *N*(*Z*) is an integer-sorted term in $FOL (\mathcal {T})$ over the variables in the set *Z*, $Z \subset X$ where *X* is the set of state variables of the transition system under consideration. *Z* typically refers to the subset of the state variables that define the parameters of the transition system; e.g. $Z = \{ \mathsf {R}{} \}$ for the Z-K proof transition system in Fig. 1, the number of blocks in a model of Path ORAM, the size of an array, etc. Typically, the variables in the set *Z* do not change after initialization. Informally stated, the counting quantifier is satisfied if a maximally large set $\varPhi {}_{\mathcal {C}}\subseteq \varPhi$, satisfying the two conditions below, has cardinality $\triangleleft ~ count$ where $count$ is the valuation of *N*(*Z*) in the initial state of every trace in $\varPhi {}_{\mathcal {C}}{}$. Those conditions are: (i) each of the traces in $\varPhi {}_{\mathcal {C}}$ are pairwise different as defined by satisfaction of $\varDelta _{\pi _{j}, \pi _{k}}$, and (ii) every trace in this set satisfies the HyperLTL formula $\psi$.

The remaining operators are standard, so we do not discuss them further and instead provide formal satisfaction semantics.

**Fig. 4.**
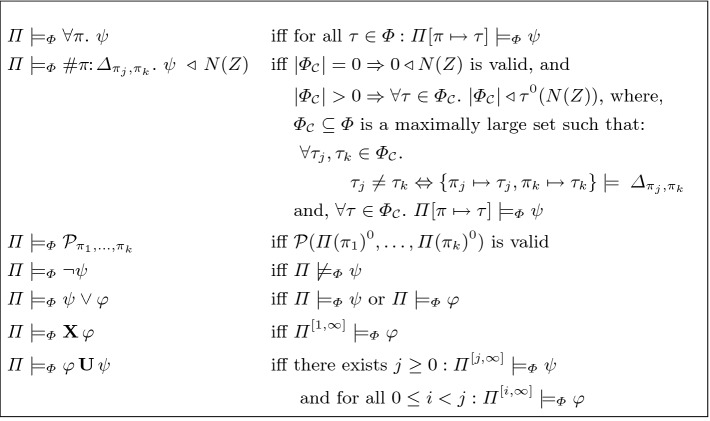
Satisfaction semantics for Quantitative HyperLTL formulas over symbolic transition systems.

**Satisfaction Semantics of Quantitative HyperLTL** The validity judgement of a property $\varphi$ by a set of traces $\varPhi {}$ is defined with respect to a trace assignment $\varPi : Vars \rightarrow \varPhi {}$. Here, $Vars$ is the set of trace variables. We use $\pi , \pi _1, \pi _2$ $, \dots$ to refer to trace variables.^4^ The partial function $\varPi$ is a mapping from trace variables to traces. We use the notation $\varPi [\pi \mapsto {\tau }_{}]$ to refer to a trace assignment that is identical to $\varPi$ except for the trace variable $\pi _{}$ which now maps to the trace ${\tau }_{}$. We write $\varPi \,\models _{\varPhi {}} \psi$ if the set of traces $\varPhi {}$ satisfies the property $\psi$ under the trace assignment $\varPi$. We will drop the subscript $\varPhi {}$ from $\models _{\varPhi {}}$ if it is clear from the context or irrelevant. The notation $\varPi ^{[i, \infty ]}$ is an abbreviation for the new trace assignment obtained by taking the suffix starting from index *i* of every trace in $\varPi$: $\varPi ^{[i, \infty ]}(\pi ) = \varPi (\pi )^{[i,\infty ]}$ for every trace $\pi \in dom (\varPi )$ where $dom (\varPi )$ is the domain of $\varPi$. We write  when $\varPi \,\models _{\varPhi {}} \psi$ is not satisfied. Satisfaction rules for HyperLTL formulas are shown in Fig. 4.

#### Definition 2 (Quantitative HyperLTL Satisfaction)

We say that the transition system $M{}$ satisfies the property $\psi$, denoted by $M{}\,\models \,\psi$ if the empty trace assignment $\emptyset$ satisfies formula $\psi$ for the set of traces $\varPhi {}_{M}$, that is $\emptyset \,\models _{\varPhi {}_{M}} \psi$.

*Additional Operators:* The above showed the minimal set of operators required in Quantitative HyperLTL. The rest of this paper will use the other standard operators such as $\wedge$ (conjunction), $\Rightarrow$ (implication), $\mathbf{F }\,$ (future/eventually) and $\mathbf{G }\,$ (globally/always) which can be defined in terms of the operators in Fig. 3.

*Well-Defined Formulas:* In order for the semantics of Quantified HyperLTL to be meaningful, we need certain semantic restrictions on the structure of QHPs.

#### Definition 3 (Well-defined QHPs)

An instance of a counting quantifier $\# \pi _{}\!\!:\!\varDelta _{\pi _{j}, \pi _{k}}.~\varphi ~\triangleleft \,N(Z){}$ is said to be well-defined if:

1. $\lnot \varDelta _{\pi _{j}, \pi _{k}}$ is an equivalence relation over the set of all traces $\varPhi {}_{}$, and
2. In every set of the traces $\varPhi {}_{\mathcal {C}}{}$ captured by the counting quantifier in the semantics shown in Fig. 4, the term *N*(*Z*) has the same valuation for all initial states: $\forall {\tau }_{i}, {\tau }_{j} \in \varPhi {}_{\mathcal {C}}{}.~{{\tau _{i}^{0}}}({N(Z)}) = {{\tau _{j}^{0}}}({N(Z)})$.

A Quantified HyperLTL formula is said to be well-defined if every instance of a counting quantifier in the formula is well-defined.

#### Example 1 (Well-defined QHPs)

The QHPs presented in the rest of this paper are all well-defined, so here we give an example of a QHP that is *not* well-defined. Consider this variant of Property 1: $\forall \pi _{0}.\# \pi _{1}\!\!:\! true .~\mathbf{G }\,(\psi _{\pi _{0}, \pi _{1}}) ~\ge \,2^\mathsf {R}- 1$. This is not a well-defined QHP because $\varDelta _{\pi _{j}, \pi _{k}}{}$ in the counting quantifier is simply $true$, and its negation is not an equivalence relation over the set of traces.

Note that condition (1) in the definition above affects $\varDelta _{\pi _{j}, \pi _{k}}$ while condition (2) places a restriction on $\varphi$. The former condition prevents double-counting of traces, while the latter ensures that the trace count is unambiguous.

The properties in our experiments require only syntactic checks to verify well-definedness. Specifically, $\varDelta _{\pi _{j}, \pi _{k}}$ is always of the form $\mathbf{F }\,(\mathcal {P}_{\pi _{j}, \pi _{k}})$ where $\mathcal {P}{}$ is of the form $\mathcal {P}({\sigma ^{}}_1, {\sigma ^{}}_2) \doteq f({\sigma ^{}}_1) \ne f({\sigma ^{}}_2)$. The negation of this is obviously an equivalence relation over the set of all traces. Secondly, our QHPs are of the form $\forall \pi _{0}.~\# \pi _{1}\!\!:\!\varDelta _{\pi _{j}, \pi _{k}}.~\varphi ~\triangleleft \,N(Z)$ where $\varphi$ enforces equality of the variables in *Z* between the traces $\pi _{0}$ and $\pi _{1}$. These two features guarantee well-definedness. In the rest of this paper, we only consider well-defined QHPs.

### Applications of QHPs in Security Specification

*Deniability:* Our first example of a quantitative hyperproperty is deniability. Suppose $obs ({\sigma ^{}})$ is a term that corresponds to the adversary observable part of the state ${\sigma ^{}}$, while $secret ({\sigma ^{}})$ corresponds to the secret component of the state ${\sigma ^{}}$. Deniability is satisfied when every trace of adversary observations can be generated by at least *N*(*Z*) different secrets. For this, we define $\delta ({\sigma ^{}}_1, {\sigma ^{}}_2) \doteq secret ({\sigma ^{}}_1) \ne secret ({\sigma ^{}}_2)$ and $\approx ^O({\sigma ^{}}_1, {\sigma ^{}}_2) \doteq obs ({\sigma ^{}}_1) = obs ({\sigma ^{}}_2)$.

$$\begin{aligned} \forall \pi _{0}.\# \pi _{1}\!\!:\!\mathbf{F }\,(\delta _{\pi _{j}, \pi _{k}}).~\mathbf{G }\,(\approx ^O_{\pi _{0}, \pi _{1}}) ~\ge \,N(Z) \end{aligned}$$

**Fig. 5.**
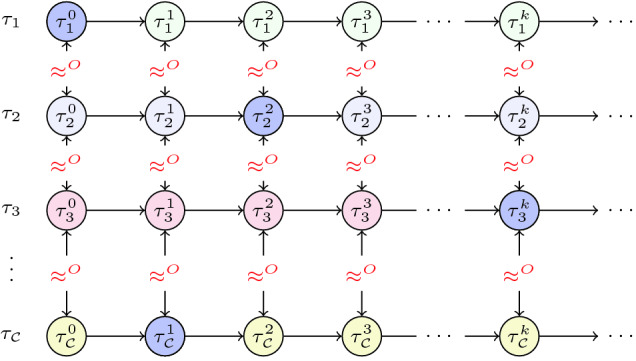
Illustrating deniability.

Figure 5 illustrates deniability. It shows a set of traces $\varPhi {}_{\mathcal {C}}{} := \{ {\tau _{1}}, {\tau _{2}}, \ldots , {\tau _{\mathcal {C}}} \}$; the circles represent the states in each trace and the secret values are shown by color of the circle. For these traces, every pair of corresponding states have the same observations: represented by $\approx ^{O}$, and every distinct pair of traces differ in the secrets. Deniability is satisfied if $|\varPhi {}_{\mathcal {C}}{}| \ge N(Z)$. Satisfaction implies that every trace of adversary observations has at least *N*(*Z*) counterparts with identical observations but different values of $secret ({\sigma ^{}})$. If we can show in a system satisfying deniability that each trace of secrets is equiprobable and *N*(*Z*) grows exponentially in some parameters of the system, then we can conclude that the system satisfies computational indistinguishability. Deniability can capture probabilistic notions of confidentiality, such as confidentiality of Path ORAM.

*Soundness:* While deniability encodes a form of confidentiality, soundness is its dual in the context of integrity. One example of soundness was given in Sect. 2.2 for the Z-K hats puzzle. Soundness is generally applicable to protocols that offer probabilistic integrity guarantees. For instance, many interactive challenge-response protocols consist of repeated rounds such that if the prover succeeds in all rounds, the verifier can be convinced with a high probability that the prover is not cheating. This can be viewed as a QHP stating that for every trace in which a dishonest prover tricks a verifier into accepting an invalid proof, there are at least *N*(*Z*) other traces with different prover responses in which the cheating is detected. As usual, we require that traces be uniformly sampled from a finite set in order to state soundness as a QHP.

Soundness is stated as $\forall \pi _{0}.\# \pi _{1}\!\!:\!\mathbf{F }\,(\delta _{\pi _{j}, \pi _{k}}).~\mathbf{G }\,(\psi _{\pi _{0}, \pi _{1}}) ~\ge \,N(Z)$. The relation $\delta$ is defined as two states having different prover responses. $\psi$ requires the challenge-response protocol to fail in $\pi _{1}$ if it succeeded in $\pi _{0}$ and also that the system parameters (the variables in *Z*) be identical between $\pi _{0}$ and $\pi _{1}$.

**Summarizing QHP Specification:** These examples demonstrate that QHPs have important applications in security verification. They capture probabilistic notions of both confidentiality and integrity. In particular, the following form of QHPs consisting of a single quantifier alternation seems especially relevant for security verification: $\forall \pi _{0}.~\# \pi _{1}\!\!:\!\varDelta _{\pi _{j}, \pi _{k}}.~\varphi ~\triangleleft \,N(Z){}$. Each of the examples of quantitative hyperproperties discussed in the previous subsection – deniability, soundness, as well as others like quantitative non-interference [46, 54] fit in this template. Therefore, in the rest of this paper, we focus on developing scalable verification techniques for QHPs that follow this template.

## Trace Enumerations

This section introduces the notion of a trace enumeration, which is a technique that allows us to reduce the problem of counting traces to that of counting satisfiable assignments to a formula in $FOL (\mathcal {T})$.

### Trace Enumeration Relations

We now formalize injective trace enumerations, which allow us to lower-bound the number of traces captured by a counting quantifier in a QHP.

#### Definition 4 (Injective Trace Enumeration)

Let us consider a transition system ${M{} = \langle X, Init {}(X), Tx {}(X,X') \rangle }{}$ and the relation ${\mathcal {U}(Y, {\tau }_{1}, {\tau }_{2})}$ where *Y* is a set of variables disjoint from *X*, ${\tau }_{1}$ and ${\tau }_{2}$ are traces of this transition system. Let $\forall \pi _{0}.~\# \pi _{1}\!\!:\!\varDelta _{\pi _{j}, \pi _{k}}.~\varphi ~\ge \,N(Z){}$ be a QHP where $Z \subset X$. Suppose $\mathcal {V}_{}({Y, Z})$ is a predicate over the variables in *Y* and *Z*. We say that the pair $\mathcal {V}_{}({Y, Z})$ and ${\mathcal {U}(Y, {\tau }_{1}, {\tau }_{2})}$ form an injective trace enumeration of the system $M{}$ for the QHP $\forall \pi _{0}.~\# \pi _{1}\!\!:\!\varDelta _{\pi _{j}, \pi _{k}}.~\varphi ~\ge \,N(Z){}$ iff the following conditions are satisfied:

1. For every trace ${\tau }_{0}$ in $\varPhi {}_{M{}}$ and every satisfying assignment $(\mathtt {Y}, \mathtt {Z})$ for the predicate $\mathcal {V}_{}({Y, Z})$, there exists a trace ${\tau }_{1} \in \varPhi {}_{M{}}$ which is related to the trace ${\tau }_{0}$ as per the relation $\mathcal {U}$ via this same assignment to *Y*. Further, the pair ${\tau }_{0}$ and ${\tau }_{1}$ satisfy the property $\varphi$ and the valuation of the variables in *Z* in the initial state of ${\tau }_{1}$ is equal to $\mathtt {Z}$.
  2 $$\begin{aligned} \forall&{\tau }_{0} \in \varPhi {}_{M{}}, {\mathtt {Y}}, \mathtt {Z}. ~\mathcal {V}_{}({{\mathtt {Y}, \mathtt {Z}}}) \Rightarrow&\\&\big (\exists {\tau }_{1} \in \varPhi {}_{M{}}.~ {\mathcal {U}({\mathtt {Y}}, {\tau }_{0}, {\tau }_{1})} \wedge \{ \pi _{0} \mapsto {\tau }_{0}, \pi _{1} \mapsto {\tau }_{1} \} \models \varphi \wedge {{\tau _{1}^{0}}}({Z}) = \mathtt {Z}\big )&\nonumber \end{aligned}$$
2. Different assignments to the variables in *Y* for the formula $\mathcal {V}_{}({Y, Z})$ enumerate different traces in ${\mathcal {U}(Y, {\tau }_{0}, {\tau }_{1})}$, where “different” means satisfaction of $\varDelta _{\pi _{j}, \pi _{k}}$.
  3 $$\begin{aligned} \forall&{\tau }_{0}, {\tau }_{1}, {\tau }_{2} \in \varPhi {}_{M{}}, \mathtt {Y}{}_1, \mathtt {Y}{}_2, \mathtt {Z}{}.~&\\&~~\mathcal {V}_{}({\mathtt {Y}{}_1, \mathtt {Z}}) \wedge \mathcal {V}_{}({\mathtt {Y}{}_2, \mathtt {Z}}) \wedge \mathtt {Y}{}_1 \ne \mathtt {Y}{}_2&\Rightarrow \nonumber \\&~~{\mathcal {U}(\mathtt {Y}{}_1, {\tau }_{0}, {\tau }_{1})} \wedge {\mathcal {U}(\mathtt {Y}{}_2, {\tau }_{0}, {\tau }_{2})} \wedge {{\tau _{1}^{0}}}({Z}) = \mathtt {Z}{} \wedge {{\tau _{2}^{0}}}({Z}) = \mathtt {Z}{}&\Rightarrow \nonumber \\&~~\{ \pi _{j} \mapsto {\tau }_{1}, \pi _{k} \mapsto {\tau }_{2} \} \models \,\varDelta _{\pi _{j}, \pi _{k}}\nonumber \end{aligned}$$

If $\mathcal {V}$ and $\mathcal {U}$ form an injective trace enumeration $M{}$ for the property $\forall \pi _{0}.~\# \pi _{1}\!\!:\!\varDelta _{\pi _{j}, \pi _{k}}.~\varphi ~\ge \,N(Z){}$, then for every trace ${\tau }_{0}$, there exist at least as many traces satisfying the counting quantifier as there are satisfying assignments to *Y* in $\mathcal {V}_{}({Y, Z})$. This is made precise in the following lemma.

#### Lemma 1

[Trace Count Lower-Bound] If $\mathcal {V}_{}({Y, Z})$ and ${\mathcal {U}(Y,{\tau }_{1},{\tau }_{2})}$ form an injective trace enumeration of the system $M{}$ for the QHP $\forall \pi _{0}.~\# \pi _{1}\!\!:\!\varDelta _{\pi _{j}, \pi _{k}}.~\varphi ~\ge \,N(Z){}$ and if ${\#}{Y}.\,{\mathcal {V}_{}({Y, Z})}$ is finite for all assignments to *Z*, then $M{}\,\models \,\forall \pi _{0}.\# \pi _{1}\!\!:\!\varDelta _{\pi _{j}, \pi _{k}}.~\varphi ~\ge \,{\#}{Y}.\,{\mathcal {V}_{}({Y, Z})}$.

#### Example 2 (Injective Trace Enumeration)

Let $\mathsf {P}_0[1], \dots , \mathsf {P}_0[\mathsf {R}{}]$ be a trace of correct responses for some particular sequence of challenges for our running example. Consider the array $\mathsf {Y}[1], \mathsf {Y}[2], \dots , \mathsf {Y}[\mathsf {R}]$ where each $\mathsf {Y}[j] \in \{0, 1\}$. $\mathsf {Y}$ is a boolean array of size $\mathsf {R}{}$, and $\mathsf {Y}[i]=1$ means that the prover gives an incorrect response to the challenge in round *i*. We can define the predicate $\mathcal {V}$ as follows.

4 $$\begin{aligned} \mathcal {V}_{}({\mathsf {Y},\mathsf {R}{}}) \doteq \;&\big (\exists i.~ 1 \le i \le \mathsf {R}\wedge \mathsf {Y}[i] \ne 0\big ) \wedge \big (\forall i.~(i < 1 \vee i > \mathsf {R}) \Rightarrow \mathsf {Y}[i] = 0\big ) \end{aligned}$$

The above definition ensures that at least one response is incorrect. Notice that for every assignment to $\mathsf {Y}$ except the assignment of all zeros, the trace of responses defined by $\forall j.~\mathsf {P}_1[j] = \mathsf {P}_0[j] \oplus \mathsf {Y}[j]$ (where $\oplus$ is exclusive or) corresponds to a valid trace of the system and satisfies the counting quantifier in Property 1. Specifically, every such response from the prover is incorrect and will result in the protocol failing. We can use the above facts to define the relation $\mathcal {U}$ as follows:

5 $$\begin{aligned} {\mathcal {U}(\mathsf {Y}, {\tau }_{1}, {\tau }_{2})} \doteq \;&\big (\forall j.~{{\tau _{1}^{0}}}({\mathsf {P}[j]}) = {{\tau _{2}^{0}}}({\mathsf {P}[j]}) \oplus \mathsf {Y}[j]\big )&\wedge&\\&{{\tau _{1}^{0}}}({\mathsf {C}}) = {{\tau _{2}^{0}}}({\mathsf {C}}) \wedge {{\tau _{1}^{0}}}({\mathsf {R}{}}) = {{\tau _{2}^{0}}}({\mathsf {R}{}}) \wedge ({{\tau _{1}^{\mathsf {R}{}}}}({S}) \Rightarrow \lnot {{\tau _{2}^{\mathsf {R}{}}}}({S}))&\nonumber \end{aligned}$$

The pair $\mathcal {V}$ and $\mathcal {U}$ form an injective trace enumeration for the system *M* (defined in Fig. 1) for the Property 1. This is because different $\mathsf {Y}$’s will result in different prover responses for the same challenges. By Lemma 1, we can conclude that Property 1 is satisfied if ${\#}{\mathsf {Y}}.\,{\mathcal {V}_{}({\mathsf {Y},\mathsf {R}{}})} \ge 2^{\mathsf {R}{}}-1$

Analogous to injective trace enumerations, it is also possible to define surjective trace enumerations that upper-bound the number of traces captured by a counting quantifier. Details of surjective trace enumerations are presented in the extended version of the paper [43].

## Model Counting

As discussed in the previous section, trace enumeration relations can bound the number of satisfying traces in a QHP. Given a QHP $\forall \pi _{0}.~\# \pi _{1}\!\!:\!\varDelta _{\pi _{j}, \pi _{k}}.~\varphi ~\triangleleft \,N(Z)$, appropriate trace enumeration predicates $\mathcal {V}_{}({Y, Z})$ and $\mathcal {U}$ can be used to derive that $\forall \pi _{0}.~\# \pi _{1}\!\!:\!\varDelta _{\pi _{j}, \pi _{k}}.~\varphi ~\triangleleft \,{\#}{Y}.\,{\mathcal {V}_{}({Y, Z}){}}$. The final step in our verification methodology is to show validity of ${\#}{Y}.\,{\mathcal {V}_{}({Y, Z})} \triangleleft N(Z)$. To that end, this section discusses our novel technique for model counting.

### Model Counting via SMT Solving

**Fig. 6.**
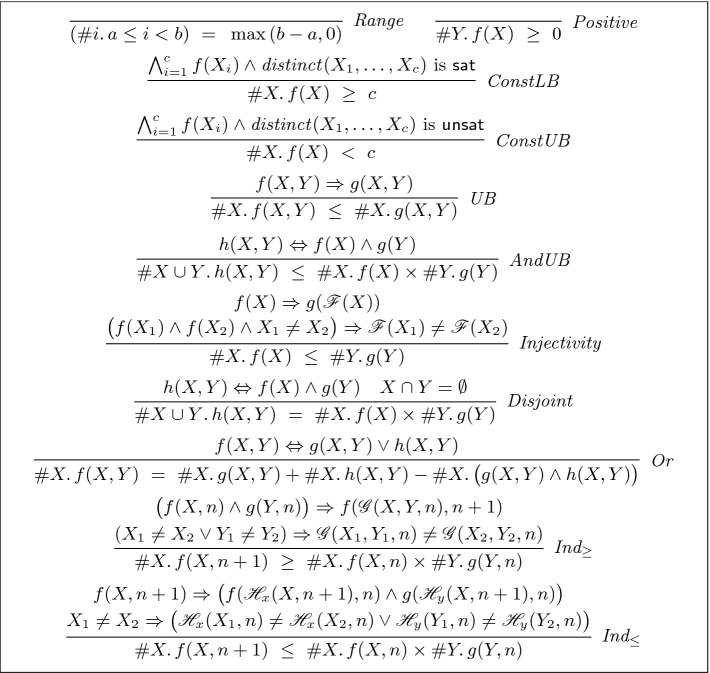
Model counting proof rules. Unless otherwise specified, premises are satisfied when the formula is valid. Conclusions have an implicit universal quantifier.

Our approach borrows ideas from enumerative combinatorics [13, 52, 56] and introduces the inference rules shown in Fig. 6 to reason about model counts for formulas in $FOL (\mathcal {T})$. Each of the conclusions in the inference rules is a statement involving model counts of $FOL (\mathcal {T})$ formulas, while each of the premises is a formula in $FOL (\mathcal {T})$ that *does not involve model counts* and can, therefore, be checked using SAT/SMT solvers. Most of the rules are straightforward, and we do not describe them due to space constraints. The three interesting rules – $Injectivity$, $Ind_\le$ and $Ind_\ge$ – are discussed below.

*Injectivity:* This rule is based on the following idea from enumerative combinatorics. Suppose we have two sets *A* and *B*. We can show that $|A| \le |B|$ if there exists an injective function from *A* to *B*. Translating this to model counts, the set *A* in the rule corresponds to satisfying assignments to *f*(*X*), *B* corresponds to satisfying assignments to *g*(*Y*) and $\mathscr {F}$ is the injective witness function.

$Ind_\ge$*and *$Ind_{\le }$*:* Suppose the formulas *f*(*X*, *n*) and *g*(*Y*, *n*) are parameterized by the integer variable *n*. If an injective witness function $\mathscr {G}(X, Y, n)$ is able to “lift” satisfying assignments of $f(X_n,n)$ and $g(Y_n, n)$ into a satisfying assignment of $f(X_{n+1}, n+1)$, then we can conclude that the number of satisfying assignments to $f(X, n+1)$ are at least as many as the product of the number of satisfying assignments to *f*(*X*, *n*) and *g*(*Y*, *n*). $Ind _\le$ is the surjective version of this rule. It applies when a satisfying assignment to $f(X_{n+1}, n+1)$ can be “lowered” into satisfying assignments to $f(X_n, n)$ and $g(Y_n, n)$ where the values of $X_n$ and $Y_n$ are given by the witness functions $\mathscr {H}_x$ and $\mathscr {H}_y$ respectively.

### Model Counting in the Motivating Example

The definition of the predicate $\mathcal {V}$ in the motivating example is shown below.

$$\begin{aligned} \mathcal {V}_{}({\mathsf {Y},\mathsf {R}{}}) \doteq ~&\big (\exists i.~ 1 \le i \le \mathsf {R}\wedge \mathsf {Y}[i] \ne 0\big ) \wedge \big (\forall i.~((i < 1 \vee i > \mathsf {R}) \Rightarrow \mathsf {Y}[i] = 0) \big ) \nonumber \end{aligned}$$

Our task is to show ${\#}{\mathsf {Y}}.\,{\mathcal {V}_{}({\mathsf {Y}, \mathsf {R}{}})} = 2^\mathsf {R}{} - 1$. Recall that $\mathsf {Y}$ is an array of binary values (i.e. the integers 0 and 1) and consider the following predicates: $\mathcal {V}_{f}({\mathsf {Y},\mathsf {R}{}}){} \doteq ~ \big (\forall i.~(i < 1 \vee i > \mathsf {R}) \Rightarrow \mathsf {Y}[i] = 0\big )$, $\mathcal {V}_{1}({\mathsf {Y},\mathsf {R}{}}) \doteq ~ \big (\forall i.~\mathsf {Y}[i] = 0\big )$ and $\mathcal {W}(i) \doteq ~ 0 \le i < 2$. Using these definitions, the proof is as follows.

1. ($$ ConstUB $$, $$ Positive $$) $${\#}{\mathsf {Y}}.\,{\mathcal {V}_{f}({\mathsf {Y},\mathsf {R}{}}){} \wedge \mathcal {V}_{1}({\mathsf {Y},\mathsf {R}{}})} = 1$$.
2. ($$ Or $$) $${\#}{\mathsf {Y}}.\,{\mathcal {V}_{f}({\mathsf {Y},\mathsf {R}{}}){}}= {\#}{\mathsf {Y}}.\,{\mathcal {V}_{}({\mathsf {Y},\mathsf {R}{}}){}}{} + {\#}{\mathsf {Y}}.\,{\mathcal {V}_{1}({\mathsf {Y},\mathsf {R}{}}){}}{}$$.
3. ($$ ConstLB $$, $$ ConstUB $$) $${\#}{\mathsf {Y}}.\,{\mathcal {V}_{1}({\mathsf {Y},\mathsf {R}{}}){}}{} = 1$$.
4. ($$ ConstLB $$, $$ ConstUB $$) $${\#}{\mathsf {Y}}.\,{\mathcal {V}_{f}({\mathsf {Y},1}){}}{} = 2$$.
5. ($$ Ind _\le $$): $${\#}{\mathsf {Y}}.\,{\mathcal {V}_{f}({\mathsf {Y},\mathsf {R}{}}){}}{} \le {\#}{i}.\,{\mathcal {W}(i)}\times {\#}{\mathsf {Y}}.\,{\mathcal {V}_{f}({\mathsf {Y},\mathsf {R}{}-1}){}}$$.
6. ($$ Ind _\ge $$): $${\#}{\mathsf {Y}}.\,{\mathcal {V}_{f}({\mathsf {Y},\mathsf {R}{}}){}}{} \ge {\#}{i}.\,{\mathcal {W}(i)}\times {\#}{\mathsf {Y}}.\,{\mathcal {V}_{f}({\mathsf {Y},\mathsf {R}{}-1}){}}$$.
7. ($$ Range $$): $${\#}{i}.\,{\mathcal {W}(i)} = 2$$.
8. (4 – 7) imply that ${\#}{\mathsf {Y}}.\,{\mathcal {V}_{f}({\mathsf {Y},\mathsf {R}{}}){}}{} = 2\times {\#}{\mathsf {Y}}.\,{\mathcal {V}_{f}({\mathsf {Y},\mathsf {R}{}-1}){}}{}$, ${\#}{\mathsf {Y}}.\,{\mathcal {V}_{f}({\mathsf {Y},1}){}}{} = 2$, this means ${\#}{\mathsf {Y}}.\,{\mathcal {V}_{f}({\mathsf {Y},\mathsf {R}{}}){}}{} = 2^\mathsf {R}{}$.
9. (2, 3, 8) imply that $${\#}{\mathsf {Y}}.\,{\mathcal {V}_{}({\mathsf {Y},\mathsf {R}{}}){}}{} = 2^\mathsf {R}{} - 1$$.

In step 5, the witness function is $\mathscr {G}(\mathsf {Y}, \mathsf {R}{}, i) \doteq \mathsf {Y}[\mathsf {R}{} + 1 \mapsto i]$, while in step 6, they are $\mathscr {H}_{\langle \mathsf {Y}, \mathsf {R}{} \rangle }(\mathsf {Y}, \mathsf {R}{} + 1) \doteq \langle \mathsf {Y}[\mathsf {R}{} + 1 \mapsto 0], \mathsf {R}{} \rangle$ and $\mathscr {H}_i(\mathsf {Y}, \mathsf {R}{}+1) \doteq (\mathsf {Y}[\mathsf {R}+1])$.^5^ Note steps 8 and 9 are automatically discharged by the SMT solver.

## Experimental Results and Discussion

In this section, we present an experimental evaluation of the use of trace enumerations for the verification of quantitative hyperproperties.

### Methodology

We studied five systems with varying complexity and QHPs. These were modeled in the Uclid5 modeling and verification framework [44, 51], which uses the Z3 SMT solver (v4.8.6) [23] to discharge the proof obligations. The experiments were run on an Intel i7-4770 CPU @ 3.40 GHz with 8 cores and 32 GB RAM.

The verification conditions are currently manually generated from the models, but automation of this is straightforward and ongoing. The *k*-trace properties were proven using self-composition [9, 10] and induction. A number of strengthening invariants had to be specified manually for the inductive proofs. Many of the invariants are relational *and* quantified and, therefore, difficult to infer algorithmically. We note that recent work has made progress toward automated inference of quantified invariants [27, 36].

### Overview of Results

Due to limited space, we only provide a brief description of our benchmarks for evaluation and refer the interested reader to the extended version of our paper [43] for a more detailed discussion. We have also made the models and associated proof scripts available at [25]. A brief overview of the case studies follows.

**Table 1.** Verification results of models.

Benchmark	Hyperproperty	Model LoC	Proof LoC	Num. Annot	Verif. Time
Electronic purse [7]	Deniability	46	93	9	3.92 s
Password checker [29]	Quantitative non-interference	59	100	10	4.69 s
F-Y array shuffle	Quantitative information flow	86	195	96	7.38 s
ZK hats (Sect. 2.2)	Soundness	91	191	36	6.34 s
Path ORAM [48]	Deniability	587	209	142	9.74 s

1. **Electronic Purse.** We model an electronic purse, with a secret initial balance, proposed by Backes et al. [7]. A fixed amount is debited from the purse until the balance is insufficient for the next transaction. We prove a deniability property: there is a sufficient number of traces with identical attacker observations but different initial balances.
2. **Password Checker.** We model the password checker from Finkbeiner et al. [29], but we allow passwords of unbounded length *n*. We prove quantitative non-interference: information leakage to an attacker is $\le$ $n$ bits.
3. **Array Shuffle.** We implement a variant of the Fisher-Yates shuffle. We chose this because producing random permutations of an array is an important component of certain cryptographic protocols (e.g., Ring ORAM [40]). We prove a quantitative information flow property stating that all possible permutations are indeed generated by the shuffling algorithm.
4. **ZK Hats.** We prove soundness of the zero-knowledge protocol in Sect. 2.
5. **Path ORAM.** Discussed in Sect. 6.3.

The properties we prove on these models and the results of our evaluation are presented in Table 1 which shows the size of each model, the number of lines of proof code (this is the code for self-composition, property specification, etc.), the number of verification annotations (invariants and procedure pre-/post-conditions) and the verification time for each example. Once the auxiliary strengthening invariants are specified, the verification completes within a few seconds. This suggests that the methodology can scale to larger models, and even implementations. The main challenge in the application of the methodology is the construction of the trace enumeration relations, associated witness functions, and the specification of strengthening invariants. Each of these requires application-specific insight. Since most of our enumerations and invariants are quantified, some of the proofs also required tweaking the SMT solver’s configuration options (e.g. turning off model-based quantifier instantiation in Z3).

### Deniability of Path ORAM

In this section, we discuss our main case study: the application of trace enumerations for verifying deniability of server access patterns in Path ORAM [48], a practical variant of Oblivious RAM (ORAM) [33]. ORAMs refer to a class of algorithms that allow a client with a small amount of storage to store/load a large amount of data on an untrusted server while concealing the client access pattern from the server. Path ORAM stores encrypted data on the server in an augmented binary tree format. Each node stores *Z* data blocks, referred to as *buckets* of size *Z*. Additionally, the client has a small amount of local storage called the *stash*. The client maintains a secret mapping called the *position map* to keep track of the path where a data block is stored on the server. Each entry in the position map maps a client address to a leaf on the server. Path ORAM maintains the invariant that every block is stored somewhere along the path from the root to the leaf node that the block is mapped to by the position map.

**Deniability of Server Access Patterns in Path ORAM:** We formulate security of access patterns in Path ORAM as a deniability property stating that for every infinitely-long trace of server accesses, there are $(\mathsf {numBlks}- 1)!$ traces of client accesses with identical server observations but different client requests.

6 $$\begin{aligned} \forall \pi _{0}.~\#\pi _{1}: \mathbf{F }\,(\delta {}_{\pi _{j}, \pi _{k}}).~ \mathbf{G }\,(\psi _{\pi _{0}, \pi _{1}})~\ge ~(\mathsf {numBlks}- 1)! \end{aligned}$$

The binary predicate $\delta$ imposes the requirement that the client’s request are different in each of the traces captured by the counting quantifier, and the condition in $\psi$ states that all the traces captured by the counting quantifier have the same observable access pattern as $\pi _{0}$.

**Verification of Deniability in Path ORAM:** To verify the QHP stated in Eq. 6, for every trace of server accesses we need to generate $(\mathsf {numBlks}-1)!$ traces of client requests that produce the same server access.

**Fig. 7.**
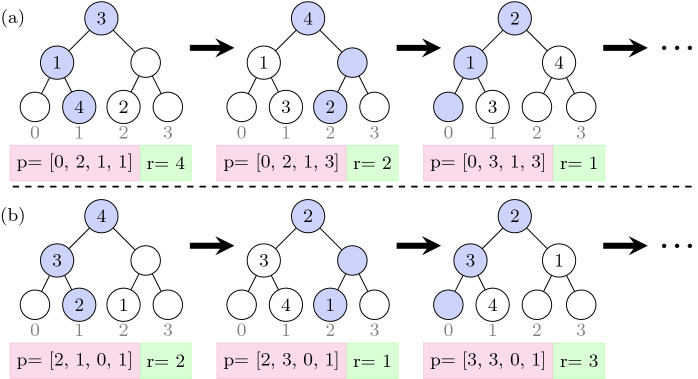
Path ORAMs satisfying the counting quantifier of Eq. 6, where, *p* represents the position map indexed from 1 and *r* is the client’s request.

Suppose we have Path ORAM (a) that is initialized with some position map. Now consider the Path ORAM (b) with the same number of blocks, but with an initial position map that is a derangement of the position map of (a).^6^ The key insight is that ORAM (b) can simulate an identical server access pattern as ORAM (a) by appropriately choosing a different client request that maps to the same leaf that is being accessed by (a) and then updating the position map identically as (a). This is shown in Fig. 7, which shows two Path ORAMs that produce identical server access patterns but service different client requests.

The above insight leads to a trace enumeration where two traces are related via $\mathcal {U}$ if their position maps are derangements of each other, the client accesses are permuted as per the derangement while all other parameters of the ORAM are identical. We use this to prove Property 6. Further details are given in [43].

## Related Work

**Hyperproperties:** Research into secure information flow started with the seminal work of Denning and Denning [24], Goguen and Meseguer [32] and Rushby [42]. The self-composition construction for the verification of secure information flow was introduced by Barthe et al. [10]. Clarkson and Schneider [21] introduced the class of specifications called hyperproperties. Clarkson and colleagues also introduced HyperLTL and HyperCTL$^*$ [19], which are temporal logics for specifying hyperproperties, while verification algorithms for these were introduced by Finkbeiner and colleagues in [30]. Cartesian Hoare Logic [47] was introduced by Sousa and Dillig and enables the specification and verification of hyperproperties over programs as opposed to transition systems. A number of subsequent efforts have studied hyperproperties in the context of program verification [5, 26, 45, 53].

**Quantitative Information Flow:** Quantitative hyperproperties build on the rich literature of quantitative information flow (QIF) [3, 17, 20, 34, 46]. The QIF problem is to quantify (or bound) the number of bits of secret information that is attacker-observable. Certain notions of QIF can be expressed as QHPs. It is important to note QHPs can express security specifications (e.g., soundness) that are not QIF. Yasuoka and Terauchi studied QIF from a theoretical perspective and showed that it could be expressed as hypersafety and hyperliveness [54]. Approaches based on QIF measures such as min-entropy [46], Shannon entropy [18] etc. have also been applied in the context of static analysis [38].

**Quantitative Hyperproperties:** Quantitative Cartesian Hoare Logic (QCHL) enables verification of certain quantitative properties of programs [16]. QHPs are more expressive than QCHL, the latter counts events within a trace (e.g. memory accesses), while QHPs count the number of traces satisfying certain conditions.

The most closely related work to ours is of Finkbeiner et al. [29] who introduced Quantitative HyperLTL over Kripke structures. They also introduced a verification algorithm for this logic that is based on maximum model counting. However, their algorithm does not scale to reasonable-sized systems, and experiments from their paper show that the approach times out when checking an 8-bit leak in a password checker (using 8-bit passwords). We differ from their work in three important ways. First, our properties are defined over symbolic transition systems rather than Kripke structures. This allows modeling and verification of QHPs over infinite-state systems. Second, our bounds are symbolic, which enables us to express bounds as functions of transition system parameters. Finally, our definition of Quantitative HyperLTL is also more expressive. It is not possible to convert our QHPs into (non-quantitative) HyperLTL formulas with *k*-traces for any fixed value of *k*.

**Verification of ORAMs:** In concurrent work with ours, Barthe et al. [11] and Darais et al. [22] have introduced specialized mechanisms to prove security of ORAMs. Barthe et al. [11] introduced a probabilistic separation logic (PSL) that (among other things) can be used to reason about the security of ORAMs. Unlike QHPs, PSL does not permit quantitative reasoning about probabilities of events and also does not (yet) support machine-checked reasoning. Darais et al. [22] introduce a type system that enforces obliviousness; they use this type system to implement a tree-based ORAM. Note that QHPs can express specifications other than obliviousness, and obliviousness need not necessarily be a QHP.

## Conclusion

Quantitative hyperproperties are a powerful class of specifications that stipulate the existence of a certain number of traces satisfying certain constraints. Many important security guarantees, especially those involving probabilistic guarantees of security, can be expressed as quantitative hyperproperties. Unfortunately, verification of quantitative hyperproperties is a challenging problem because these specifications require simultaneous reasoning about a large number of traces of a system. In this paper, we introduced a specification language, satisfaction semantics, and a verification methodology for quantitative hyperproperties. Our verification methodology is based on reducing the problem of counting traces into that of counting the number of assignments that satisfy a first-order logic formula. Our methodology enables security verification of many interesting security protocols that were previously out of reach, including confidentiality of access pattern accesses in Path ORAM.

## Footnotes

1 Our experiments mostly use the AUFLIA theory which allows arrays, uninterpreted functions, and linear integer arithmetic.

2 We note there is an implied universal quantifier here. To be precise, we must write $\forall Y.~{\#}{X}.\,{F(X, Y)}= {\#}{X}.\,{G(X, Y)} + {\#}{X}.\,{H(X, Y)}$.

3 A counting quantifier over Kripke structures was introduced by Finkbeiner et al. [29]. Our definition is slightly different and a detailed comparison is deferred to Sect. 7.

4 Note the distinction between trace variables denoted by $\pi _{1}, \pi _{2}$, etc. and traces which are denoted by ${\tau }_{1}, {\tau }_{2}$, etc.

5 The notation $arr [i \mapsto v]$ denotes an array that is identical to $arr$ except for index *i* which contains *v*.

6 A **derangement** of a set is a permutation of the elements of the set such that no element appears in its original position.
